# MKL1/2 and ELK4 co-regulate distinct serum response factor (SRF) transcription programs in macrophages

**DOI:** 10.1186/1471-2164-15-301

**Published:** 2014-04-23

**Authors:** Lan Xie

**Affiliations:** 1 Medical Systems Biology Research Center, Department of Biomedical Engineering, Tsinghua University School of Medicine, Beijing 100084, China; 2 Department of Cellular and Molecular Medicine, University of California, San Diego, 9500 Gilman Drive, La Jolla, CA 92093-0651, USA; 3 National Engineering Research Center for Beijing Biochip Technology, 18 Life Science Parkway, Beijing 102206, China

**Keywords:** SRF, MKL1, MKL2, ELK4, Gene expression array, ChIP-seq

## Abstract

**Background:**

Serum response factor (SRF) is a widely expressed transcription factor involved in multiple regulatory programs. It is believed that SRF can toggle between disparate programs of gene expression through association with different cofactors. However, the direct evidence as to how these factors function on a genome-wide level is still lacking.

**Results:**

In the present study, I explored the functions of SRF and its representative cofactors, megakaryoblastic leukemia 1/2 (MKL1/2) and ETS-domain protein 4 (ELK4), during fungal infection challenge in macrophages. The knockdown study, combined with gene expression array analysis, revealed that MKL1/2 regulated SRF-dependent genes were related to actin cytoskeleton organization, while ELK4 regulated SRF-dependent genes were related to external stimulus responses. Subsequent chromatin immunoprecipitation coupled with massively parallel sequencing (ChIP-seq) suggested that many of these regulations were mediated directly in *cis*.

**Conclusions:**

I conclude that SRF utilizes MKL1/2 to fulfill steady state cellular functions, including cytoskeletal organization, and utilizes ELK4 to facilitate acute responses to external infection. Together, these findings indicate that SRF, along with its two cofactors, are important players in both cellular homeostasis and stress responses in macrophages.

## Background

Macrophages are an important subset of innate immune cells implicated in host defense against bacterial and viral infections. Three of their major functions include phagocytosis and degradation of foreign pathogens, clearance of apoptotic or necrotic host cells, and rapid generation of effector cytokines that orchestrate immune responses. These different functions of macrophages are likely regulated by distinct transcription programs.

Serum response factor (SRF) is a member of the MADS (*M*cm1, *A*gamous, *D*eficiens, *S*RF) transcription factor family [1] and is abundantly expressed in a variety of cell types. Deletion of *Srf* is embryonic lethal [2]. Conditional deletion of *Srf* revealed its essential functions in many tissues, including skeletal, cardiac and smooth muscle cells [3,4], neurons [5], and thymocytes [6,7]. At the cellular level, these phenotypes can be attributed to impaired expression of different sets of SRF target genes. SRF-regulated immediate early genes (IEGs), such as *c-fos*, early growth response 1 (*Egr1*) and *junB*, are important for cell growth and proliferation [8-10], while SRF-regulated *troponin*, vinculin (*Vcl*), and *SM22α* participate in muscle-specific contractile functions [9,11].

With high affinity and specificity, homodimeric SRF binds to a consensus *cis*-element known as a CArG box, with the sequence of CC(A/T)_6_GG [12,13]. SRF regulates diverse programs of gene expression through its association with different accessory factors. The most well-studied cofactors of SRF include members of the ternary complex factor (TCF) and myocardin families [9] of transcription factors. The TCF family of proteins includes ELK1, ELK3 (also known as SAP2, Net) and ELK4 (also known as SAP1). TCFs respond to mitogen-activated protein kinase (MAPK) signaling initiated by serum or growth factors [14] and is involved in the regulation of IEGs. TCFs interact with SRF and simultaneously bind to an ETS binding site adjacent to the CArG box. Together, the CArG box and the ETS site are recognized as the serum response element (SRE) [15]. Myocardin family members include myocardin, megakaryoblastic leukemia 1 (MKL1, also known as MAL, MRTF-A and BSAC) and MKL2 (also known as MRTF-B). Myocardin is strictly expressed in cardiac and smooth muscle cells, while MKL1 and 2 exhibit broader expression patterns [16]. MKLs connect actin dynamics with SRF activation [17] and can regulate muscle-specific contractile genes in response to actin polymerization [18]. Unlike TCFs, MKL1 and MKL2 do not bind DNA sequences directly [19]; instead, they associate with SRF to regulate the transcription of target genes. SRF interaction with MKLs and TCFs is mutually exclusive [20,21], but recently, MKLs were reported to regulate the expressions of some IEGs in response to serum induction [22,23]. However, little is known about how these two families coordinate with SRF on a genome-wide level.

Macrophages express a variety of toll-like receptors (TLRs) on their cell surface that enable recognition of molecules, known as pathogen-associated molecular patterns (PAMPs) [24], that are broadly expressed by pathogens, but not by the host. Zymosan, a yeast cell wall derivative, induces inflammatory responses in macrophages through binding to the dectin-1 receptor or TLR2/6 heterodimers [25]. The function of SRF in macrophages had not been investigated until our previous studies revealed that SRF regulates both general and cell type-restricted cytoskeletal gene expression programs in these cells [26]. Interestingly, we also found that the SRF/MADS (CArG box) motif is highly enriched in the promoters of zymosan-induced genes and that SRF indirectly modulates type I interferon signaling in macrophages [27]. Overall, these studies suggest a possible role of SRF in the immune responses of macrophages.

With recent advances in parallel high throughput sequencing, I sought to map out the genome-wide occupancy of SRF and its cofactors, MKL1/2 and ELK4. In this study, RNA interference was used to knock down SRF, MKL1/2, and ELK4, followed by genome-wide expression profiling analysis and ChIP-seq experiments to identify the target genes and direct binding sites of these cofactors. Together, I demonstrate that the two SRF cofactor families, TCFs and MKLs, regulate distinct subsets of SRF target gene programs during fungal infection in macrophages.

## Results

### SRF is required for macrophage responses to zymosan treatment

In order to identify the transcription factors that are involved in gene regulation in response to fungal infection in macrophages, I stimulated primary macrophages with zymosan for 1 h and harvested RNA for microarray analysis. Motif analysis on the promoters of genes shown by the array to be induced by zymosan identified the SRF/MADS (CArG box) motif as one of the most highly enriched motifs [27] (Additional file 1: Figure S1A). This is in accordance with the fact that SRF functions as an immediate responder to external stimuli such as growth factors or serum. Interestingly, *Srf* transcription was increased 2-fold after 1 h of zymosan treatment, but returned to baseline after 6 h of treatment (Additional file 1: Figure S1B), suggesting that SRF activation is an immediate early phase response. To evaluate if this change in mRNA expression had a functional effect, I transfected RAW264.7 cells with a reporter construct containing 3 tandem SRF-binding *cis*-elements (3×CArG). After 1 h of treatment with zymosan, I found an approximately three-fold increase in reporter activity (Additional file 1: Figure S1C). Next, I evaluated the impact of knocking down *Srf* in zymosan treated elicited mouse peritoneal macrophages by using microarray analysis [27]. Considering that the transcription of *Srf* was induced quickly and that the SRF motif was enriched in the early phase of zymosan treatment, I decided to focus the rest of my study on cells treated with ligand for short periods of time. To identify SRF-dependent genes, I filtered the microarray results to isolate the set of transcripts whose expressions were reduced by more than 1.5 fold with SRF-specific siRNA after 1 h of zymosan treatment. The resulting subset contained 264 non-redundant downregulated genes and 203 upregulated genes (full list in Additional file 2: Table S1), which indicates that SRF can be induced by zymosan stimulation and that it is an important contributor to macrophage responses to external stimuli.

**Figure 1:**
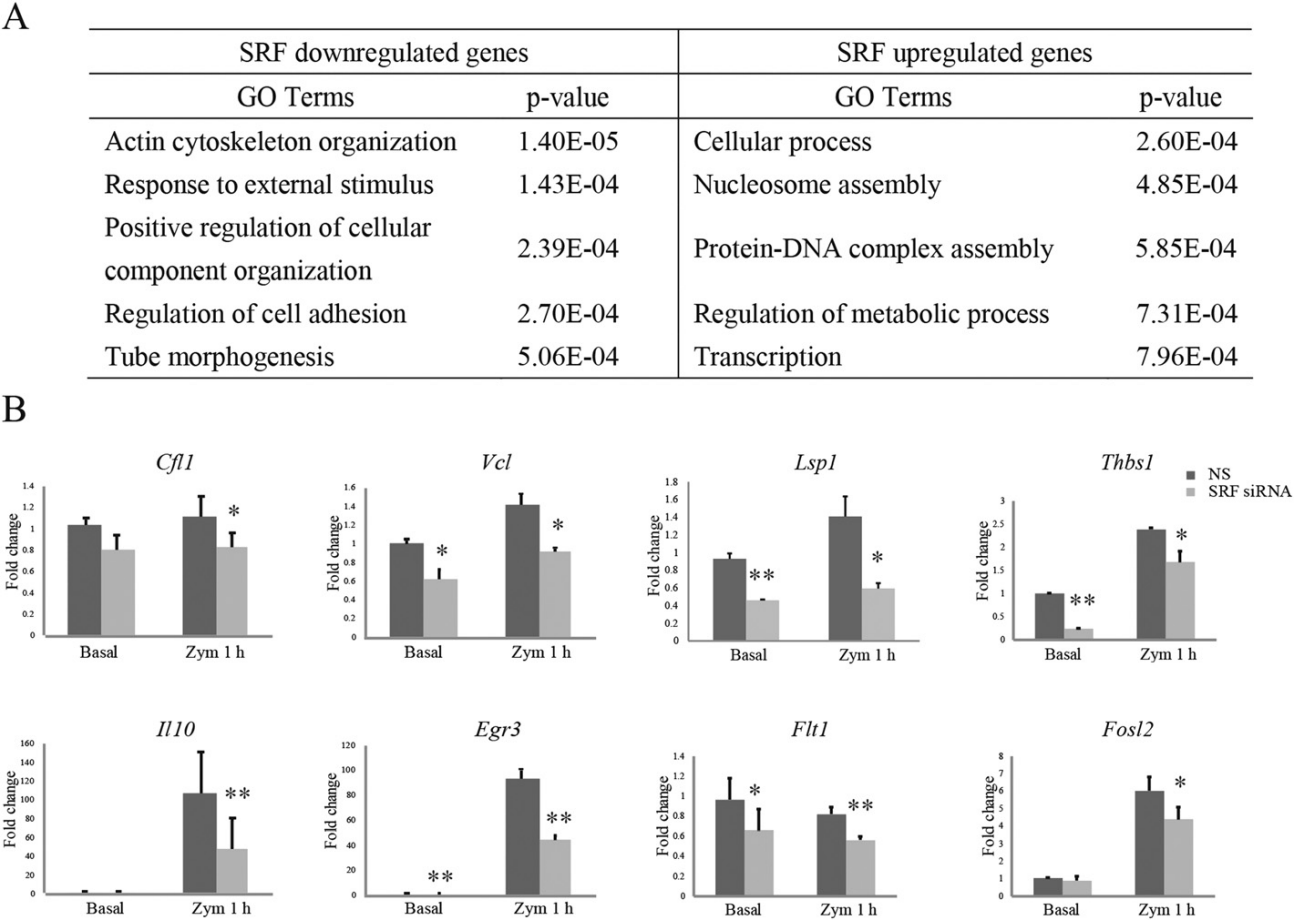
**SRF is required for macrophage function in response to zymosan stimulation. (A)** Gene ontology analysis for biological process annotations of downregulated and upregulated SRF dependent genes after zymosan treatment for 1 h. **(B)** mRNA expression profiles of the indicated representative SRF dependent genes were analyzed by Q-PCR. Thioglycollate-elicited macrophages were transfected with nonspecific (NS) or specific siRNAs for SRF and treated with or without zymosan for 1 h. **p < 0.01 *vs.* NS; *p < 0.05 *vs.* NS. Target gene expression is represented as the average fold change compared to NS siRNA transfected samples of at least three independent experiments. Error bars represent standard deviations.

To investigate which biological functions the SRF target genes are involved in, I performed gene ontology (GO) analysis of the SRF regulated genes using the DAVID Functional Annotation Tool. The top 5 functional annotations are shown in Figure 1A (full results in Additional file 3: Table S2, Part I). The most significantly enriched item was “actin cytoskeleton organization”, which is a function of SRF that has been elucidated in other cell types and in our previous study in quiescent macrophages [26]. It is noteworthy that some genes within this subgroup , such as *Coro1a*[28], *Lsp1*[29] and *Lcp1*[30], are preferentially expressed in cells that are of myeloid or hematopoietic origin and regulate macrophage specific functions such as phagocytosis or migration. Moreover, other GO terms that are closely related to macrophage specific functions were also significantly enriched such as “regulation of cell adhesion” (including *Cd24a, Cited2, Csf1, Itga6, MMP14, Srf, Thbs1* and *Tgm2*) and “response to external stimulus” (including *Cd24a, Bat5, Hps1, MEFV, Arg1, F13A1, C1QB, CFP, Coro1a, ENTPD1, GPR77, Igfbp4, Igta6, Lcp1, Lsp1, Mmp13, Mmp14, Mest, USF2, Thbs1* and *Il10*).

I validated the expression changes of some representative target genes by quantitative PCR (Q-PCR). As expected, several groups of target genes were confirmed as SRF dependent, including: (1) general actin cytoskeleton related genes such as cofilin1 (*Cfl1*) and *Vcl,* and the migration related gene, lymphocyte-specific protein 1 (*Lsp1*); (2) adhesion related genes like thrombospondin 1 (*Thbs1*); (3) genes related to stimulus responses such as *Il10* and *Egr3*; and (4) genes related to other functions, including *fms-*related tyrosine kinase 1 (*Flt1*) and fos-like antigen 2 (*Fosl2*) (Figure 1B). All of these results indicate that SRF is required for intact expression of genes closely related to macrophage functions.

GO analysis of the genes upregulated by SRF knockdown under zymosan treated conditions resulted in the identification of more generic terms such as “cellular process”, “nucleosome assembly” and “regulation of metabolic process” (Figure 1A, full results in Additional file 3: Table S2, Part II). Besides regulating the transcription of target genes, SRF is known to regulate some miRNAs [31-33] associated with altered expression of mRNAs and proteins. The genes upregulated upon SRF knockdown could potentially be downstream targets of SRF through the regulation of miRNAs. Using Ingenuity Pathway Analysis, I compiled a list of 10 overrepresented miRNAs for the 203 upregulated genes, which included miR-16-5p, miR-489, miR-205-5p, miR-101, miR-291a-3p, miR-204-5p, miR-33, miR-615-3p, miR-424-3p and miR-10a-5p. Although there is no current literature linking any of these miRNAs to SRF, it would be interesting to test, in future studies, whether these miRNAs are actually novel targets of SRF.

### MKL1/2 and ELK4 regulate different subsets of genes in zymosan treated macrophages

Previous studies of SRF in muscle cells suggested that SRF participates in different regulatory programs by associating with different transcription cofactors. This mechanism enables SRF to toggle between regulating cell proliferation and growth and orchestrating actin cytoskeletal and contractile homeostasis. I next sought to investigate accessary factors utilized for SRF function in response to zymosan stimulation in macrophages. I focused on two well-known SRF associated cofactor families: the myocardin and TCF families of transcription cofactors.

MKL1 and MKL2, share many redundant functions [23] and are both highly expressed in primary macrophages. Unlike *Srf*, mRNA levels of *Mkl1* and *Mkl2* stay constant after zymosan treatment (Additional file 4: Figure S2A). Because of their many redundancies, I used an siRNA pool targeting both *Mkl1* and *Mkl2* to knock down their expressions simultaneously to better assess their role in macrophages (Additional file 4: Figure S2A, MKL1/2 will denote both MKL1 and MKL2 in the following text). *Elk4* is the dominantly expressed member of the TCF family in primary macrophages (Additional file 4: Figure S2B). Similar to *Mkl1/2*, *Elk4* expression does not change during zymosan treatment (Additional file 4: Figure S2B). Transfection of macrophages with *Elk4*-specific siRNAs significantly reduced *Elk4* expression compared with control siRNA transfected samples (Additional file 4: Figure S2C). I also confirmed that the knockdown effect is specific for *Elk4* and that mRNA expression for *Elk1* and *Elk3* were unaffected (Additional file 4: Figure S2C). Following confirmation of the siRNA knockdown efficiencies, I performed gene expression array analysis on my samples to compare the gene expression changes in *Mkl1/2* and *Elk4* knockdown cells *versus* control siRNA transfected cells after zymosan treatment for 1 h.

Using 1.5 fold reduction as a cutoff, I identified a total of 309 genes that were MKL1/2 dependent (full list in Additional file 5: Table S3) and 265 genes that were ELK4 dependent (full list in Additional file 6: Table S4). These results indicate that the absence of these cofactors would have an impact on zymosan induced gene expression in macrophages.

**Figure 2:**
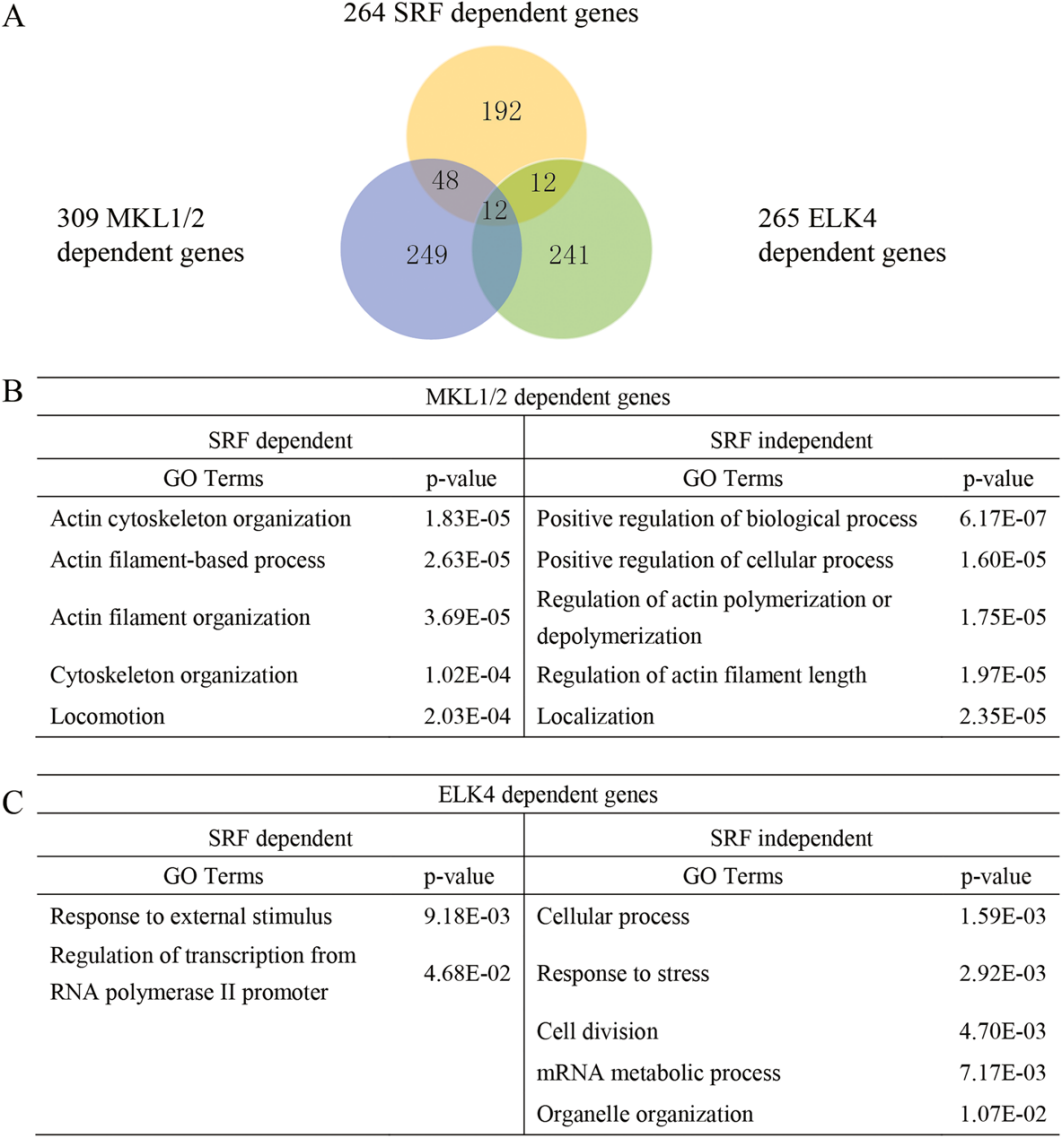
**MKL1/2 and ELK4 regulate the transcription of different gene sets in zymosan induced macrophages. (A)** Venn diagram indicating the relationships between SRF dependent genes with respect to MKL1/2 and ELK4 dependence. **(B)** Gene ontology analysis for biological process annotations of the SRF dependent or independent MKL1/2 target genes after zymosan treatment for 1 h. **(C)** Gene ontology analysis for biological process annotations of the SRF dependent or independent ELK4 target genes after zymosan treatment for 1 h.

To check if MKL1/2 contributes to SRF related functions in macrophages, I compared the 309 MKL1/2 target genes with the 264 SRF target genes. This analysis identified 60 MKL1/2 target genes that were also dependent on SRF for their expression (Figure 2A). GO analysis of these 60 genes identified the term of “actin cytoskeleton organization” as the top hit. When I performed GO analysis on the MKL1/2 target genes that were independent of SRF expression, I found that “positive regulation of biological process” ranked as the first significant term (Figure 2B). Taken together, these results suggest that MKL1/2 could coordinate with SRF to regulate cytoskeleton related target genes, but may also have other generic functions independent of SRF.

By comparing the 265 ELK4 target genes with the 264 SRF target genes, I identified 24 genes that were dependent on both ELK4 and SRF for their expression (Figure 2A). GO analysis of these 24 genes identified two significantly enriched terms: “response to external stimulus” and “regulation of transcription from RNA polymerase II promoter”. This result indicates that ELK4 is involved in a different aspect of SRF function than MKL1/2. The fact that ELK4 dependent genes are enriched for the GO term related to stress responses in gene ontology analysis is consistent with our finding that besides the SRF motif, the ELK4 motif is also highly enriched in the promoters of zymosan-induced genes in primary macrophages (Additional file 1: Figure S1A). Since the majority of ELK4 dependent target genes are not dependent on SRF for expression, ELK4 may have some SRF independent functions in macrophages. Similar GO analysis on the SRF independent ELK4 target genes showed the term “cellular process” shifted to the top, followed by “response to stress” and “cell division”, *etc.* (Figure 2C).

Taken together, these findings support the view that MKL1/2 and ELK4 are involved in distinct functions of SRF in zymosan treated macrophages. Specifically, MKL1/2 is involved in actin cytoskeleton organization, ELK4 contributes to establishing responses to external stresses, and both sides constitute important functions of SRF in macrophages. Moreover, I found that both MKL1/2 and ELK4 have roles that are independent of SRF and that they may perform these roles independently or in coordination with other transcription factors. Additional work will be necessary to explore how SRF and these cofactors work on the genome directly and also to identify other potential cofactors.

### MKL1/2 and ELK4 dependent target genes show different responses to zymosan induction

**Figure 3:**
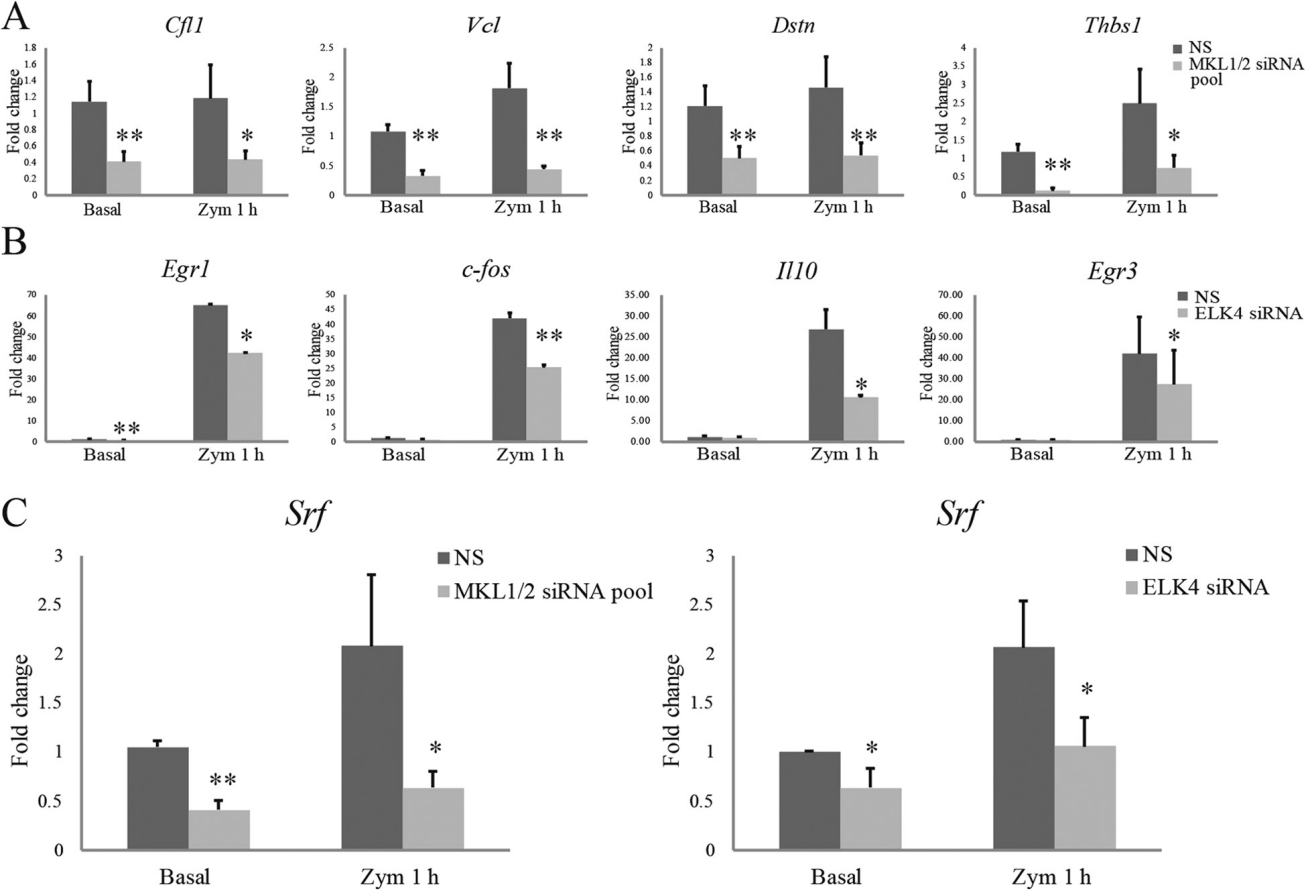
**Expression profiles of MKL1/2 and ELK4 dependent target genes in response to zymosan treatment. (A)** and **(B)** Expressions of MKL1/2 dependent genes (*Cfl1, Dstn, Vcl* and *Thbs1*) and ELK4 dependent genes (*Il10, c-fos, Egr1* and *Egr3*) were analyzed by Q-PCR. Thioglycollate-elicited macrophages were transfected with NS or specific siRNAs for MKL1/2 or ELK4, as indicated, and treated with or without zymosan for 1 h. **(C)***Srf* mRNA expression in response to MKL1/2 or ELK4 knockdown under basal conditions or after treatment with zymosan for 1 h. Target gene expression is represented as the average fold change compared to NS siRNA transfected samples of three independent experiments. **p < 0.01 *vs.* NS; *p < 0.05 *vs.* NS. Error bars represent standard deviations.

Similar to the SRF target genes, I also validated representative target genes of MKL1/2 and ELK4 by Q-PCR. I verified the MKL1/2 dependence of a subset of genes with functional annotations linked to actin cytoskeleton organization, including *Cfl1, Dstn*, *Vcl,* and *Thbs1.* All four of these genes are highly expressed in macrophages in the basal state. While the former two genes were not regulated by zymosan, the latter two were slightly upregulated by zymosan treatment. Expression of all 4 genes was reduced 30-60% by MKL1/2 knockdown (Figure 3A). Moreover, ELK4 expression was not required for the expression of genes related to actin cytoskeleton organization such as *Vcl* and *Cnn2* (Additional file 4: Figure S2D).

I also verified the ELK4 dependence of a subset of stimulus response genes, including *Egr1, c-fos, Il10,* and *Egr3* (Figure 3B)*.* The expression of these four genes can be induced as much as 100 fold by zymosan, in contrast to actin related genes. Although not revealed by microarray evaluation, I found by Q-PCR that knocking down MKL1/2 also impaired the expression of some stimulus response genes such as *Egr3* and *Il10* (Additional file 4: Figure S2E).

### Generation of a BLRP-MKL1-BirA stable cell line for ChIP-seq analysis in RAW264.7 macrophages

While knockdown experiments combined with mRNA expression analysis helped to identify the genes whose expressions are dependent on SRF, MKL1/2 and ELK4, it could not tell me whether these dependencies are through direct transcriptional regulation or through a secondary effect. It has been reported (and our expression data confirms) that SRF regulates the expression of itself [34,35] and that this effect is dependent on both MKL and TCF cofactors [14,18,36]. Thus, it is possible that knocking down MKL1/2 and ELK4 could cause reduced expressions of some target genes through reducing the expression of *Srf*. As shown in Figure 3C, knocking down MKL1/2 reduced the expression of *Srf* more than 50%, under both basal and zymosan treated conditions (p < 0.001 and p = 0.017, respectively). Knocking down ELK4 showed a similar effect (p = 0.036 and 0.013, respectively). This result is consistent with previous reports that there is an MKL and TCF-dependent positive feedback loop for SRF-dependent autoregulation [14,18,36].

To identify the direct targets of SRF and its cofactors on a genome-wide level, I performed ChIP followed by high throughput sequencing of the enriched DNA fragments (ChIP-seq). MKL1 and MKL2 do not bind DNA sequences directly [19]; instead, they associate with SRF to regulate the transcription of target genes. As the binding affinity of MKL1/2 to the DNA fragment is relatively weak, conventional ChIP assay with MKL1 antibody failed to provide sufficient enrichment on known target genes in macrophages (data not shown). To this end, I generated a BLRP-tagged MKL1 expression vector and transfected it into RAW264.7 cells to generate a stable cell line exogenously expressing BLRP-MKL1 (BLRP-MKL1-BirA stable cell line). A TEV-based ChIP strategy was then performed for MKL1 using this stable cell line, followed by sequencing analysis. I note here that SRF and ELK4 directly bind DNA, so ChIP-seq using commercial antibodies against endogenous proteins were possible in the MKL1 overexpressed BirA stable cell line.

Stable clones with highly expressed BLRP-MKL1 were identified and selected by RT-qPCR with primers targeting the BLRP sequence (Additional file 7: Figure S3A). *Blrp* expression was approximately 4000 times higher in the stable cell line compared to parental BirA cells and the *Blrp* mRNA can be further induced as much as 10 fold following 1 h of zymosan treatment. MKL1 expression levels in this stable clone were twice as high as in the BirA parental cell line under basal conditions and were induced 7.5 fold after 1 h of zymosan treatment (Additional file 7: Figure S3A). The high expression of MKL1 protein was also confirmed by western blot (Additional file 7: Figure S3B).

### Global localization of SRF, MKL1 and ELK4 in macrophages

**Figure 4:**
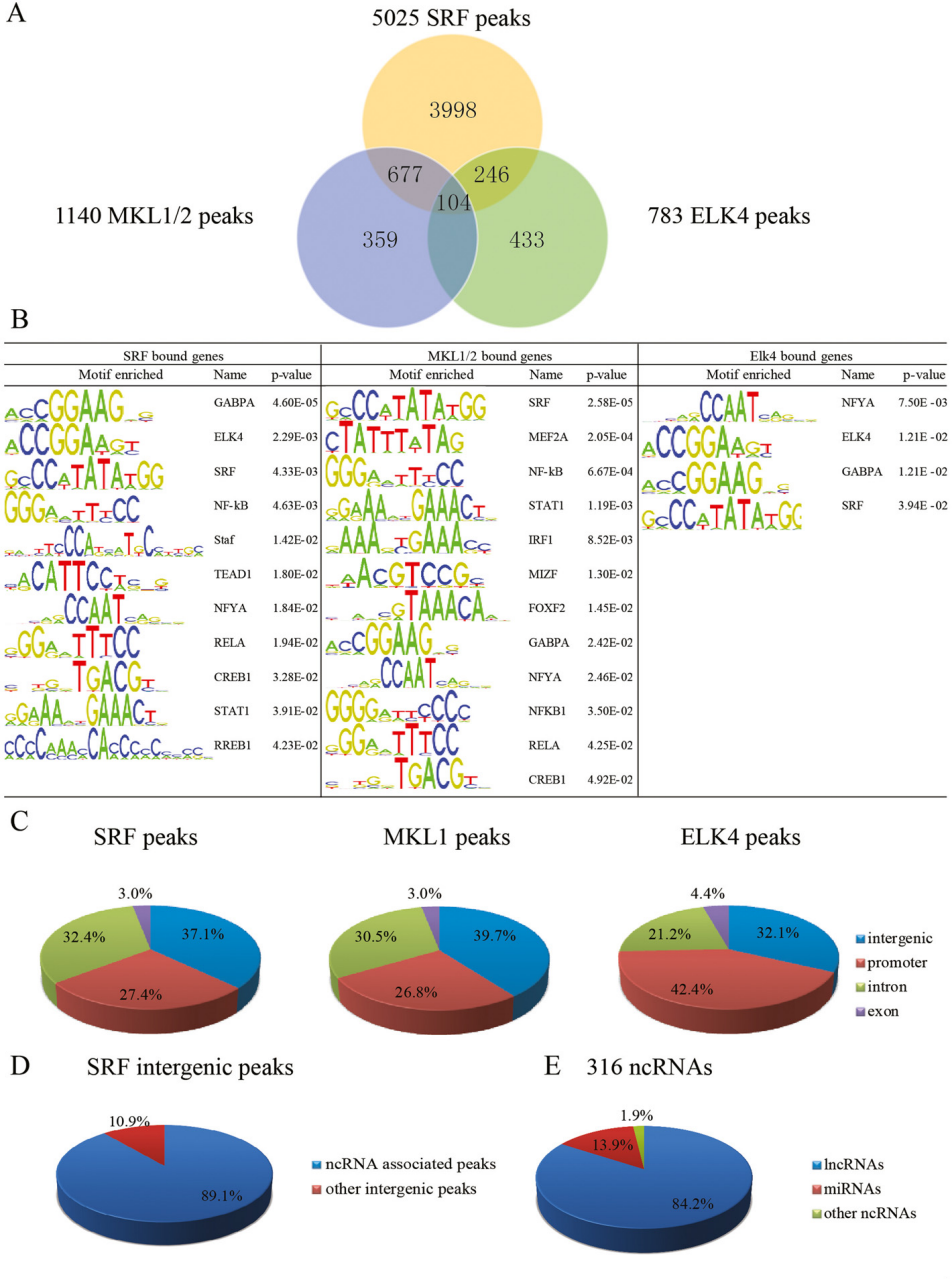
**Genomic analysis of SRF, MKL1 and ELK4 ChIP-seq peaks in macrophages. (A)** Venn diagram of the overlap between SRF, MKL1, and ELK4 peaks identified by ChIP-seq experiments in BLRP-MKL1-BirA stable cells. **(B)** Motif enrichment analysis of all significant SRF, MKL1 and ELK4 bound genes. **(C)** Genomic location annotation analysis of significant SRF, MKL1 and ELK4 ChIP-seq peaks, as indicated (>20 tag counts). **(D)** The percentage of ncRNAs associated with SRF bound intergenic peaks. **(E)** The classification of ncRNAs associated with SRF bound intergenic peaks.

All the ChIP-seq assays were performed in zymosan treated BLRP-MKL1-BirA stable cells. Using a false discovery rate of 0.1%, I identified a total of 5025 SRF-specific peaks, 1140 MKL1-specific peaks and 783 ELK4-specific peaks (Full lists are provided in Additional file 8: Table S5, Additional file 9: Table S6 and Additional file 10: Table S7, respectively). Each of these peaks were then annotated to the closest RefSeq gene observed. Among the 5025 SRF-bound peaks, I found 781 and 350 peaks that were also associated with MKL1 and ELK4 binding, respectively. Furthermore, 104 SRF peaks were associated with both MKL1 and ELK4 (Figure 4A). There were also a number of SRF associated peaks that did not overlap with MKL1 and ELK4 binding sites, indicating that there may be other cofactors utilized by SRF. For MKL1, the majority of the peaks (68.5%, 781 out of 1140) were associated with SRF. However, the percentage for ELK4 association with SRF peaks was relatively lower (44.7%, 350 out of 783), indicating that ELK4 may have other functions independent of SRF and could have other co-regulators [37].

Motif analysis of the 5025 nearest genes associated with SRF peaks by oPOSSUM recovered many transcription factor binding sites (TFBSs). As expected, SRF and ELK4 were among the top hits, together with GABPA, NFκB, Staf, TEAD1, NFYA, RELA, CREB1, STAT1 and RREB1 (Figure 4B). Some of the overrepresented TFs have been reported as cofactors of SRF already, including NFκB, TEAD1, NFYA and RELA [38-42]. My study also revealed additional potential cofactors of SRF, such as GABPA, Staf, CREB1, STAT1 and RREB1. The most highly enriched motif for MKL1 bound genes was SRF, followed by MEF2A, NFκB, STAT1, IRF1, MIZF, FOXF2, GABPA, NFYA, RELA and CREB1 (Figure 4B). Most of the overrepresented TFBSs of MKL1 peaks coincided with those of SRF (including NFκB, STAT1, GABPA, NFYA, RELA and CREB1), suggesting that MKL1 might be involved in the same complex with SRF and other cofactors. However, there is also the possibility that MKL1 might interact with the novel cofactors, independently of SRF, to regulate specific target genes.

For ELK4 bound genes, ELK4 and SRF motifs were significantly enriched, together with NFYA and GABPA (Figure 4B). NFYA has been predicted to interact with both ELK4 and ELK1 [43]. GABPA, another ETS-domain transcription factor, has been reported to show a substantial overlap in promoter occupancy with ELK1 and ELK4 [44]. The association of ELK4 with both NFYA and GABPA motifs suggests that, besides SRF, these factors could be new partners of ELK4 in macrophages and should be experimentally validated in future work.

Classification of SRF peaks with respect to the locations of genes revealed that a large fraction of SRF peaks were located in intergenic regions (37.1%) and introns (32.4%). Approximately 27.4% of the peaks were located in the vicinity of promoters (+/- 1000 bp of a transcription start site) and only 3.0% in exons (Figure 4C). This distribution pattern is consistent with our previous SRF ChIP-seq studies in quiescent primary macrophages [26]. A large fraction of the peaks seen in primary macrophages (923 peaks, 73.1% of the total SRF bound peaks) were also identified in my SRF ChIP-seq in BLRP-MKL1-BirA cells. Genomic annotation of MKL1 binding sites indicated a distribution pattern similar to SRF peaks. 39.7% of the MKL1 peaks were located in intergenic regions, 30.5% in introns, 26.8% in promoters and a very small fraction in exons (3.0%) (Figure 4C). However, the genomic distribution of ELK4 binding sites differed significantly from that of SRF and MKL1. Approximately 42.4% of the ELK4 peaks were located in promoters, 32.1% in intergenic regions, 21.2% in introns and a very small fraction in exons (4.4%) (Figure 4C). This data indicates that ELK4, unlike SRF and MKL1, may function primarily through promoter-proximal regions.

The fact that there is a difference in the number of SRF ChIP-seq peaks in intergenic regions *versus* those of ELK4, indicates that SRF participates in some unique transcription regulation mechanisms independent of its well-known cofactor, ELK4. There are two possible explanations for this phenomenon. First, SRF could function at distal enhancers for its regulated genes. This possibility was verified by our previous study showing that SRF binds together with PU.1 at distal sites to regulate cell-type specific cytoskeletal genes using an enhancer-based strategy [26]. Second, since many non-coding RNAs (ncRNAs) are found in intergenic regions, it is possible that SRF regulates the expression of ncRNAs. To test this notion, I examined if there are annotated ncRNA loci within 20 kb of the 1827 SRF intergenic binding sites. My *in silico* analysis showed that a significant portion (10.89%) of the intergenic peaks were associated with annotated ncRNAs (Figure 4D). A total of 316 unique ncRNAs (Additional file 11: Table S8) were associated with intergenic SRF peaks, indicating the possibility of regulation of these ncRNAs by SRF. A majority of the ncRNAs (84.49%, 267) identified are classified as long non-coding RNAs (lncRNAs), while 44 are miRNAs (Figure 4E). In support of the possibility of SRF regulation of ncRNAs, the previously reported SRF target miRNAs, miR-1 and miR-133, were on the miRNA list generated by my *in silico* analysis.

**Figure 5:**
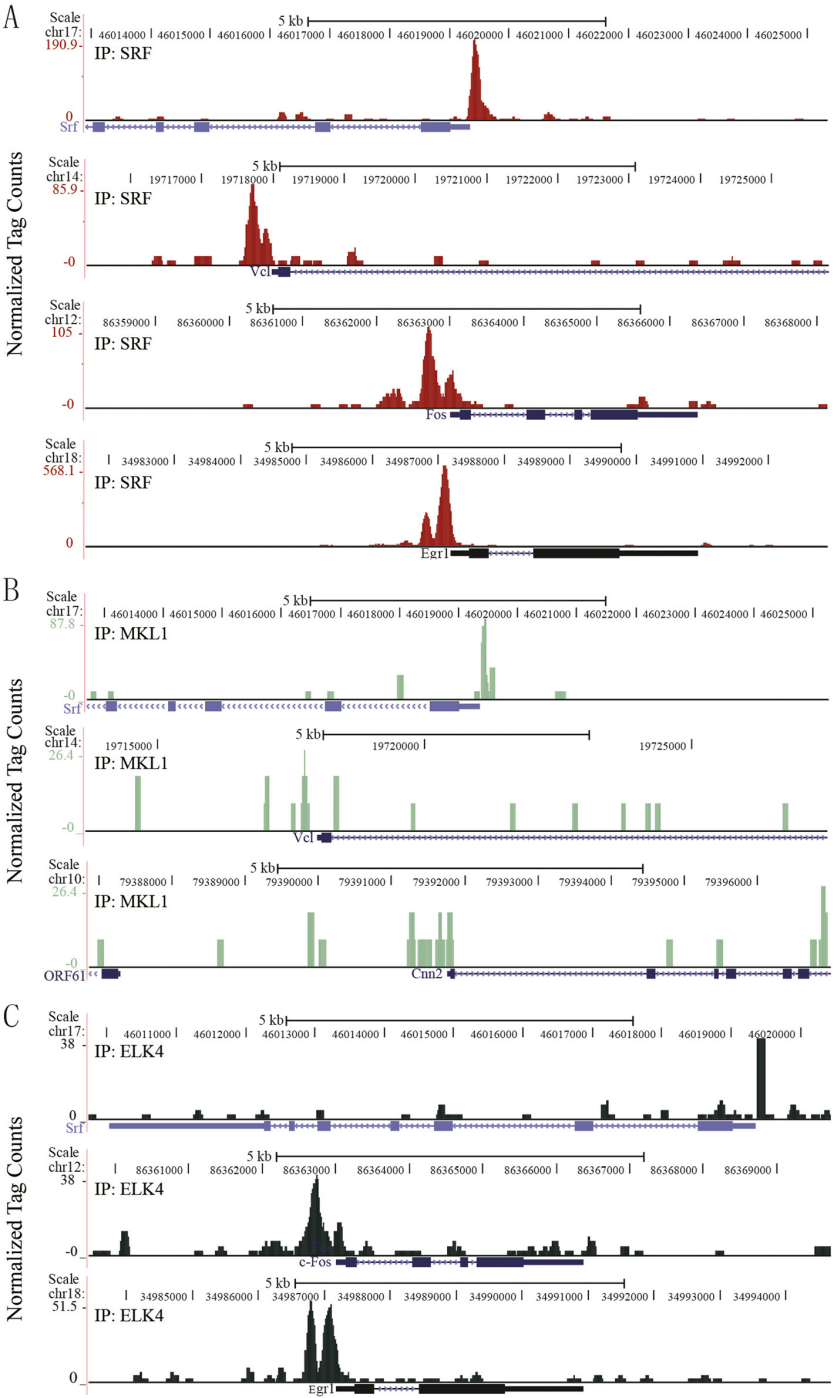
**UCSC Genome Browser images for SRF, MKL1 and ELK4 ChIP-seq peaks on representative target genes. (A)** UCSC Genome Browser image of SRF ChIP-seq data at the promoters of *Srf, Vcl, c-fos* and *Egr1*, as indicated. **(B)** UCSC Genome Browser image of MKL1 ChIP-seq data at the promoters of *Srf, Vcl* and *Cyr61*, as indicated. **(C)** UCSC Genome Browser image of ELK4 ChIP-seq data at the promoters of *Srf, c-fos* and *Egr1*, as indicated.

UCSC Genome Browser images of SRF binding to 4 of its target genes (*Srf, Vcl, c-fos* and *Egr1*) are shown in Figure 5A. Three representative MKL1 peaks associated with *Srf, Vcl* and *Cyr61* are shown in Figure 5B. Three representative ELK4 peaks on *Srf, c-fos* and *Egr1* are shown in Figure 5C.

### MKL1 and ELK4 are dedicated to different transcription programs in macrophages

**Figure 6:**
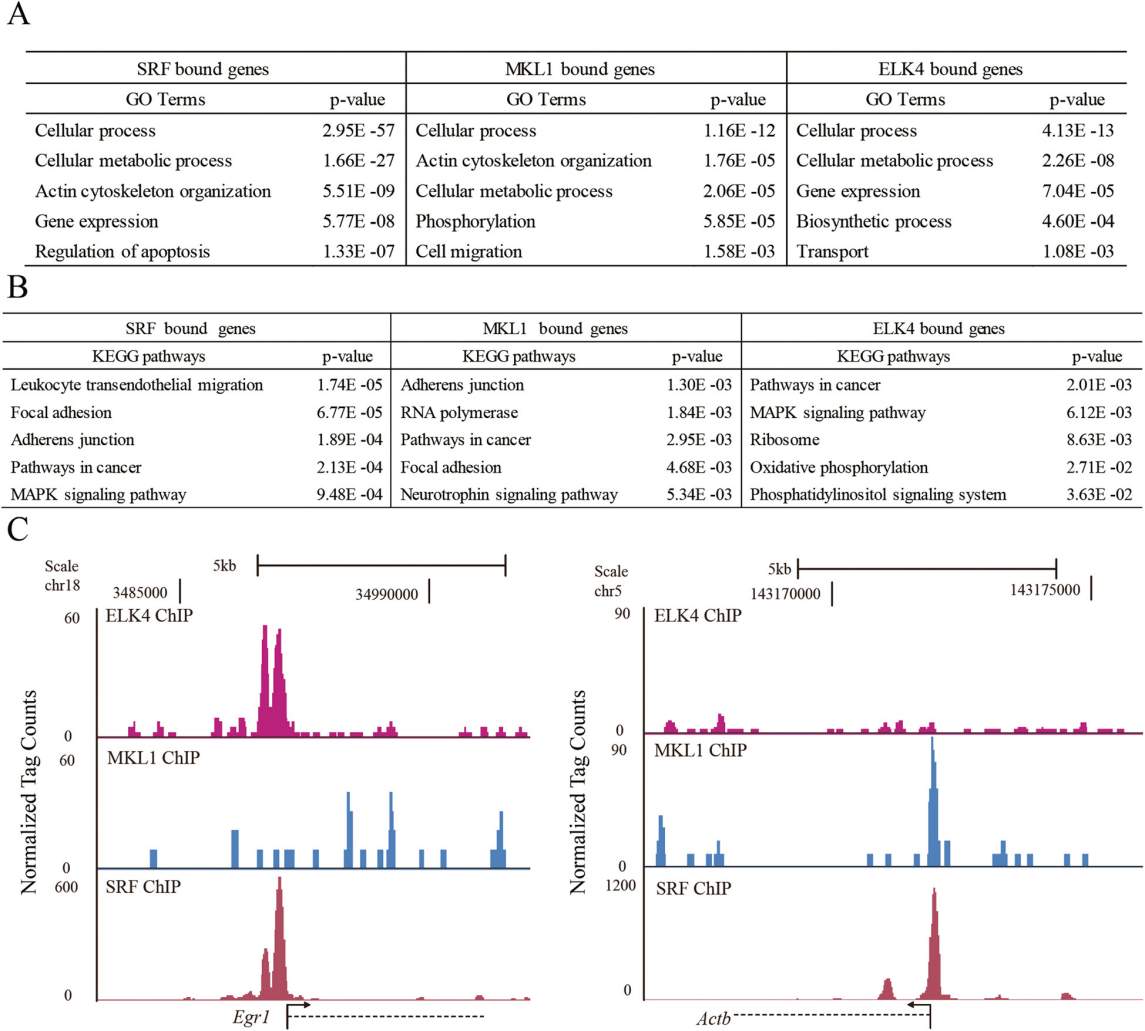
**MKL1 and ELK4 contribute to distinct programs in SRF mediated transcription regulation. (A)** Gene ontology analysis for biological process annotations of SRF, MKL1 and ELK4 peak associated genes, as indicated. **(B)** KEGG pathway annotations for SRF, MKL1 and ELK4 peak associated genes, as indicated. **(C)** UCSC Genome Browser images of SRF, MKL1 and ELK4 ChIP-seq peaks found near the *Egr1* and *Actb* genes, as indicated.

To check the functional significance of DNA binding of SRF, MKL1 and ELK4, I used the functional annotation tool, DAVID, to assess genes associated with binding peaks of SRF, MKL1 and ELK4, respectively. The top 5 most relevant GO terms of biological process for each target gene list are presented in Figure 6A (full results in Additional file 12: Table S9, Parts I, II and III). SRF associated genes were overrepresented with GO terms of “actin cytoskeleton organization” and “gene expression”. The GO term “actin cytoskeleton organization” was also selectively enriched in MKL1 associated genes. In contrast, “gene expression” appeared as one of the most highly enriched terms for ELK4 associated genes.

By using KEGG pathway analysis for the SRF, MKL1, and ELK4 bound genes, I found that MKL1 and ELK4 associated genes contribute to different pathways related to SRF. For example, SRF and MKL1 associated genes showed overlapping and enriched pathways of “focal adhesion” and “adherens junction”. In contrast, SRF associated genes were enriched in the “MAPK signaling pathway” with ELK4 associated genes. The KEGG term of “pathways in cancer” was commonly seen in the analysis of all of the 3 groups of target genes (Figure 6B, full results in Additional file 13: Table S10, Parts I, II and III). These functional differences can be illustrated by different target genes regulated by the two different cofactors. *Actb* is an example of a “focal adhesion” KEGG pathway molecule and *Egr1* is a representative “MAPK signaling pathway” molecule. Each of these genes are independently regulated by MKL1 and ELK4 as indicated by genome browser images. As shown in Figure 6C, *Actb* is an MKL1-dependent and ELK4-independent SRF target gene. In contrast, *Egr1* is bound by ELK4 and SRF, but not MKL1.

**Figure 7:**
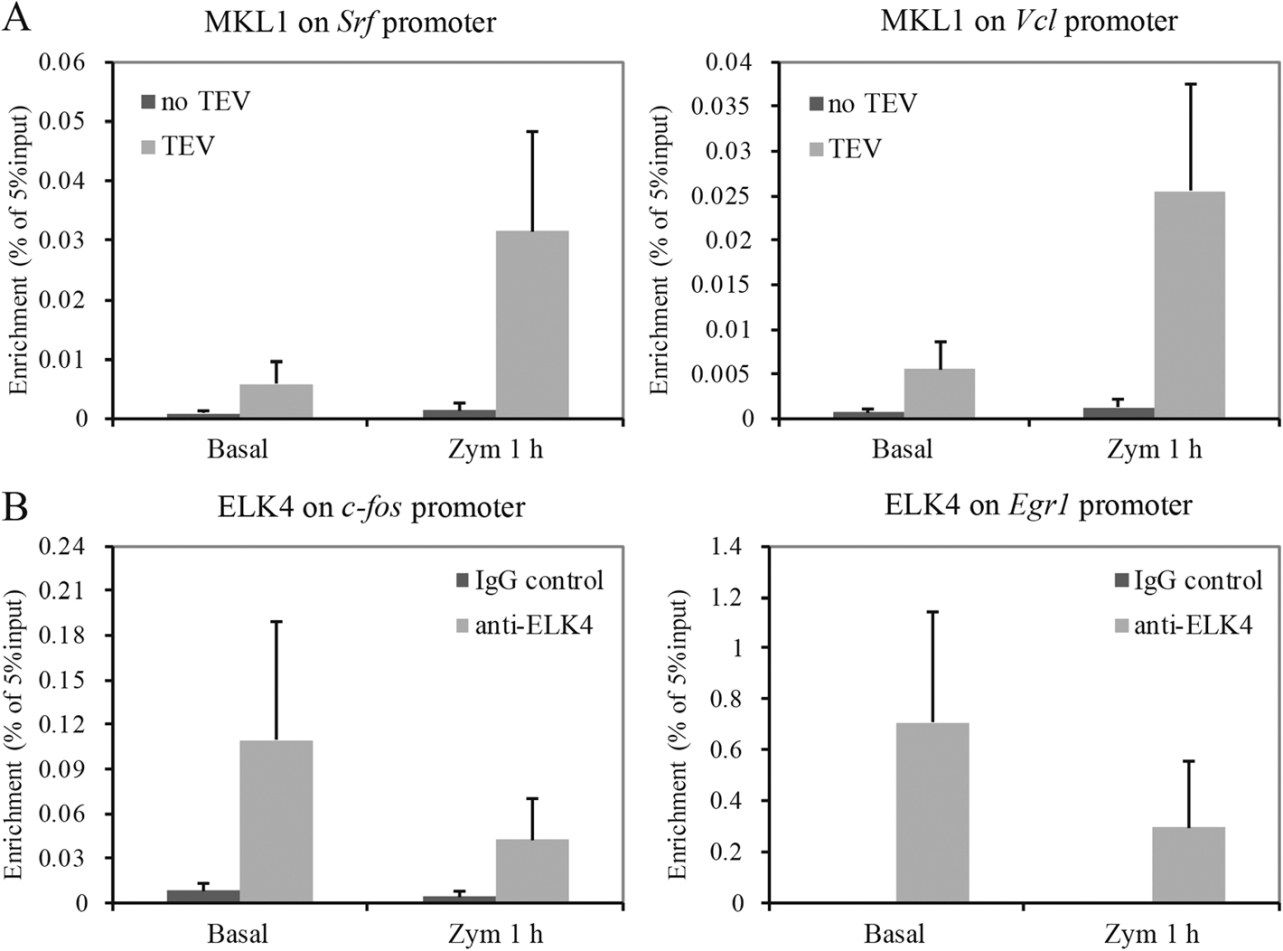
**Validation of MKL1 and ELK4 recruitment to target gene promoters following zymosan treatment. (A)** MKL1 ChIP assay was performed in BLRP-MKL1-BirA stable cells under basal conditions and after zymosan treatment for 1 h. Immunoprecipitated DNA was analyzed by Q-PCR using primers flanking the proximal promoter regions of *Srf* and *Vcl*. **(B)** Conventional ELK4 ChIP assay was performed in BLRP-MKL1-BirA stable cells under basal conditions and after zymosan treatment for 1 h. Rabbit IgG was used as a negative control. Immunoprecipitated DNA was analyzed by qPCR using primers flanking the proximal promoter regions of *c-fos* and *Egr1*.

I further confirmed the occupancy of MKL1 and ELK4 on different target genes by ChIP-qPCR and also compared the dynamic binding of MKL1 and ELK4 before and after zymosan treatment. My experiments confirmed the abundant enrichment of MKL1 on the promoters of *Srf* and *Vcl*. The observed enrichments were high under basal conditions and were further enhanced after zymosan treatment (Figure 7A). This was in accordance with the increased expression of MKL1 in response to zymosan treatment in the BLRP-MKL1-BirA cell line. Conventional ChIP analysis using primers flanking the peaks also confirmed ELK4 binding to the promoters of *c-fos* and *Egr1* (Figure 7B). Although the mRNA expressions of these two target genes can be highly induced by zymosan treatment, ELK4 occupancy levels remained constant on these two promoters.

## Discussion

In this study, I analyzed the role of SRF, and representative members of its cofactor families in macrophages, in the context of zymosan stimulation. Using a knockdown strategy coupled with gene expression array analysis, I identified target genes whose expressions were dependent on the intact expression of SRF, MKL1/2 and ELK4, respectively. Chromatin immunoprecipitation combined with high-throughput sequencing allowed the identification of direct target genes of SRF, MKL1 and ELK4. Taken together, my results indicate that MKL1 and ELK4 contribute to distinct aspects of SRF functions in macrophages.

Here, I show that SRF mediates a response to zymosan stimulation in primary macrophages. The transcription of *Srf* was increased by zymosan treatment in an early phase, similar to other highly characterized immediate early genes in the literature such as *c-fos* and *Jun*. Identification of the SRF binding element, the CArG box, as one of the most highly enriched motifs in the promoters of zymosan induced genes suggests that SRF may play important roles in zymosan responses in macrophages. Using knockdown analysis combined with gene profiling, I identified different target gene sets for SRF and members of two well-known families of SRF accessory cofactors: the myocardin family members, MKL1/2, and the TCF family member, ELK4. With regard to the effects of knocking down SRF, I found that different gene sets with macrophage specific functions were targets of SRF, including genes related to migration, phagocytosis, adhesion and responses to stimuli. In agreement with previous studies performed in other cell types, MKL1/2 regulates the expression of many genes related to actin cytoskeleton organization in macrophages. These genes showed high basal expression and little to no induction in response to zymosan treatment. In contrast, I found that ELK4 target genes are overrepresented in the GO term of “response to stress”. I validated some representative ELK4 target genes and found that the expressions of these genes were highly induced by zymosan treatment and that their inductions were heavily dependent on intact ELK4 expression. Taken together, it’s proposed that MKL1/2 and ELK4 contribute to different functions in the quiescent state compared to the context of an inflammatory response. Moreover, the microarray data revealed that both MKL1/2 and ELK4 have some SRF independent functions and that these functions may involve other novel cofactors.

One of the limitations of microarray analysis is that it is difficult to differentiate the direct target genes from the secondary ones. For example, *Srf* expression itself can be affected by knocking down MKL1/2 or ELK4. I also noticed that some ELK4 dependent target genes showed decreased expression with MKL1/2 loss, which is probably a secondary effect of SRF expression downregulation. Therefore, I performed ChIP-seq analysis to further identify direct target genes of SRF and its cofactors and to explore novel partners of the three transcription factors.

Motif analysis of the SRF bound peaks identified in my ChIP-seq experiment revealed a set of transcription factors that could be cofactors of SRF besides ELK4. Some of these factors have already been reported as cofactors of SRF. For example, SRF acts in conjunction with TEAD1 for cardiac muscle gene regulation [38,42], the interaction of SRF and NFYA has been verified *in vitro*[39], and the interaction of SRF and NFκB has been observed on the IL-2R enhancer in T lymphocytes [40]. In addition, the NFκB subunit, p65 (also known as RELA), synergizes with SRF to activate an SRE containing reporter [41]. My study also revealed several additional potential cofactors of SRF, including GABPA, Staf, CREB1, STAT1 and RREB1. An independent ChIP-seq study in Jurkat T cells found that a large fraction of SRF peaks occur within 100 bp of GABP peaks, suggesting that SRF may interact physically with GABP [45]. Although CREB has not been reported as directly interacting with SRF, CREB binding protein (CBP) has been found constitutively bound to the SRE on the *c-fos* promoter [46]. RREB1 and SRF were also both predicted to interact with the same factor, MEF2 [43]. These references provide some evidence for my hypothesis that SRF may interact with cofactors outside of the myocardin and TCF families.

Our previous ChIP-seq study of SRF in primary macrophages indicated a critical role of SRF in promoting hematopoietic-cell-restricted gene expression through binding at distal enhancers, while modulation of generally expressed genes occurred at promoters [26]. A large fraction of the SRF peaks in primary quiescent macrophages also showed up in my ChIP-seq experiment in the BLRP-MKL1-BirA cells. Moreover, I also found that SRF-binding sites are not restricted to promoter regions, but are preferentially located in intergenic regions of the mouse genome. Through mapping of the intergenic peaks to the mouse genome, I found that 10.89% of the peaks were associated with annotated ncRNAs, of which, lncRNAs constitute the major fraction. A list of miRNAs were also associated with intergenic SRF peaks, including the known SRF targets, miR-1 and miR-133 [33]. While MKL1 has a genomic distribution pattern similar to SRF, as previously reported [19], ELK4 showed a relatively larger fraction of occupancy on promoters. In particular, GABPA and NFYA motifs were found to be enriched on ELK4 bound gene promoters, suggesting a possible partnership of the two transcription factors with ELK4.

In addition to validating the MKL1 and ELK4 binding sites from my ChIP-seq experiment, I also assessed the binding of these factors in the context of zymosan stimulation. With regard to mRNA expression, MKL1 dependent genes showed high basal expression and little to no induction in response to zymosan treatment. By ChIP-qPCR, I found that MKL1 binding to the promoters of its target genes was increased after zymosan treatment. Considering the higher expression of MKL1 after zymosan treatment in the BLRP-MKL1-BirA stable cells, this enhanced occupancy may reflect an increased expression of BLRP-MKL1 protein. In contrast, ELK4 target gene mRNA levels can be highly induced by zymosan treatment. However, in the basal state, ELK4 is already bound to target genes poised for zymosan induction. I speculate that additional events, such as post-translational modification of the SRF-cofactor complex, occur upon zymosan treatment to promote the function of ELK4-SRF at these loci. Kasza, *et al.* reported similar results in which ELK1 and SRF were both found on the ZC3H12A promoter and that their binding was also not increased upon IL-1β stimulation [47]. These results indicate that transcription factor recruitment may not be a key regulatory event in response to inflammatory stimuli.

## Conclusions

In summary, this is the first study to examine the functions of SRF and its key cofactors simultaneously on a genome-wide level through both ChIP-sequencing and transcriptome analysis. The overall view of the genome-wide locations of SRF, MKL1 and ELK4 has been constructed and the results suggest that SRF utilizes MKL1 to fulfill steady state cellular functions, including cytoskeletal organization, and utilizes ELK4 to facilitate acute responses to external infection. Previous studies of SRF largely focused on skeletal, cardiac and smooth muscle cells and neurons. This project sheds light on the functions of SRF, MKL1 and ELK4 in macrophages, indicating their potential roles for both cellular homeostasis and stress responses in immune cells.

## Methods

### Cell culture and transient transfection

Thioglycollate (BD biosciences) -elicited, peritoneal macrophages were prepared as previously described [48] from 6–8 week old, male, C57BL/6 mice (Harlan). For RNAi experiments, 0.75 million primary macrophages were transfected with nonspecific (NS) control or SMARTpool siRNAs (100 nM, Dharmacon) directed against *Srf, Mkl1, Mkl2* and *Elk4* using the Deliver X transfection reagent (Panomics) according to the manufacturer’s instructions. RAW 264.7 were transiently transfected with a 3xCArG luciferase reporter using Superfect reagent (Qiagen) and luciferase activities were measured using the luciferase assay system (Promega). Cells were treated with or without zymosan A (1 mg/mL, Sigma-Aldrich) as stated in each experiment.

### Generation of BLRP-MKL1-BirA cell lines

To achieve rapid and high affinity purification of MKL1 for ChIP-sequencing assay, I generated a BLRP (biotin ligase recognition peptide)-TEV-Flag-MKL1 expression vector containing a puromycin resistance gene. BLRP is a high affinity substrate for the *E. coli* biotin ligase, BirA, which biotinylates a lysine residue within the peptide [49,50]. A RAW264.7 cell line that stably expresses BirA under neomycin selection has been previously described [49]. The BLRP-MKL1 expression vector was transfected into the BirA expressing RAW264.7 stable cell line and stable clones expressing both BirA and BLRP-tagged MKL1 were identified by puromycin and neomycin double selection.

### RNA isolation and Q-PCR

Total RNA (isolated by RNeasy kit, Qiagen) was prepared from untreated or zymosan treated primary macrophages and digested with DNase I according to the manufacturer's instructions. cDNA was synthesized from 1 μg of total RNA using Superscript III (Invitrogen) reverse transcriptase according to the manufacturer's instructions. Quantitative real-time PCR (Q-PCR) analysis was performed using 1 μl of cDNA template, 50 ng of each gene-specific primer, and SYBR greenER master mix (Invitrogen) on a 7300 real-time PCR system (Applied Biosystems). Primer sequences are available upon request.

### Gene expression profiling

For the *Mkl1/2* double knockdown samples, 0.5 μg of purified RNA per sample was labeled using the LRILAK PLUS, two color low RNA input Linear Amplification kit and hybridized to Whole Mouse Genome Microarray 4 × 44 K 60 mer slides (Agilent) according to the manufacturer’s instructions. Slides were scanned using the Agilent GZ505B Scanner. For *Elk4* knockdown samples, 100 ng of purified RNA per sample was reverse transcribed using M-MLV (Takara Chemicals) and amplified by an *in vitro* transcription based method. The Cyanine 3 (Cy3) or Cyanine 5 (Cy5) labeled cDNA was hybridized to the CapitalBio Mouse Genome Oligo Array (CapitalBio) for 16 h at 42°C. Arrays were scanned with a LuxScan™ 10 K-A confocal scanner (CapitalBio), and the data were extracted with GenePix pro4.0 software (Axon Instruments).

### Gene ontology (GO) analysis and motif analysis

GO analysis was performed using the web-based functional-annotation tool, DAVID (http://david.abcc.ncifcrf.gov/home.jsp). Gene ontology terms were considered significant if they had a p-value of less than 0.05. The oPOSSUM human/mouse single site analysis tool (http://www.cisreg.ca/oPOSSUM/) was used to identify motifs of 12-bps in length that were overrepresented in different groups of target sequences [51]. Motifs with a Z-score of more than 5 and a Fisher Score of less than 0.05 were regarded as significantly enriched. Terms presented here are sorted by their respective Fisher scores. Analysis of the overrepresented miRNAs upstream of target genes was performed using Ingenuity Pathway Analysis (Ingenuity Systems, http://www.ingenuity.com).

### Chromatin immunoprecipitation (ChIP) assay

ChIP assays against MKL1 were performed using the BLRP-MKL1-BirA cell line with methods similar to those previously described in [50]. Briefly, cells were crosslinked with formaldehyde then chromatin was fragmented into 200–300 bp pieces by sonication and subjected to high affinity purification with Dynabeads® MyOne™ Streptavidin T1 (Life technologies). The beads were subjected to AcTEV Protease (Life Technologies) digestion to release the tagged MKL1 proteins complexed with interacting genomic DNA, leaving non-specific proteins bound to the beads. The MKL1-DNA elution without TEV digestion was used as a background control. Elutions from TEV digestion were reverse-crosslinked by heating, and DNA was recovered and purified.

ChIP assays against SRF and ELK4 were performed in the BLRP-MKL1-BirA cell line with methods similar to those previously described in [26]. Immunoprecipitations were performed by incubating with 2.5 μg of anti-SRF antibody (sc-335, Santa Cruz Biotechnology) or anti-ELK4 antibody (sc-13030, Santa Cruz Biotechnology) overnight at 4°C. Protein-DNA complexes were recovered using 50 μl of blocked ImmunoPure Protein A Agarose (Pierce) and then washed, eluted, digested and purified. Rabbit preimmune serum was used as a control for non-specific binding.

### High-throughput ChIP sequencing (ChIP-seq) and data analysis

Purified ChIP DNA was adapter ligated and PCR amplified according to the manufacturer’s instructions (Illumina). High-throughput sequencing was performed on an Illumina Genome Analyzer and short reads (first 23 to 25 bp) were mapped to the mouse reference genome. Tag counts for each experiment were normalized to 10^7^ specifically mapped tags. Sequences with tag counts of more than 20 were regarded as binding peaks. To determine SRF peaks, SRF ChIP-seq data was compared to a background data set where SRF ChIP-seq was performed in SRF knockout primary macrophages. Only those peaks with tag counts at least 4-fold more than the tag counts in SRF knockout cells were considered specific. Peaks were visualized by preparing custom tracks using the UCSC Genome Brower (http://www.genome.ucsc.edu/) based on the protocol previously described [26]. Peaks were assigned to specific genes by proximity to the nearest transcription start site. To search for associated ncRNAs, sequences +/-20 kb away from target peaks were extracted and mapped to annotated ncRNAs based on UCSC mm9 and NCBI build37.

## Competing interests

The authors declare that she has no competing interests.

## Authors’ contributions

LX designed the study, carried out all the experiments, and drafted the manuscript.

## Supplementary Material

Additional file 1: Figure S1 SRF functions in the early phase of zymosan induction in macrophages. Click here for file

Additional file 2: Table S1 Genes that are downregulated (Part I) or upregulated (Part II) more than 1.5 fold in SRF knockdown primary macrophages after zymosan treatment for 1 h. Click here for file

Additional file 3: Table S2 Part I: Gene Ontology results (Biological process annotations, p < 0.05) for SRF downregulated genes (downregulated more than 1.5 fold with SRF knockdown in zymosan treated macrophages). Part II: Gene Ontology results (Biological process annotations, p < 0.05) for SRF upregulated genes (upregulated more than 1.5 fold with SRF knockdown in zymosan treated macrophages). Click here for file

Additional file 4: Figure S2 Knockdown of MKL1/2 and ELK4 has an impact on zymosan treated macrophages. Click here for file

Additional file 5: Table S3 Genes that are downregulated more than 1.5 fold in MKL1 and MKL2 double knockdown primary macrophages after zymosan treatment for 1 h. Click here for file

Additional file 6: Table S4 Genes that are downregulated more than 1.5 fold in Elk4 knockdown primary macrophages after zymosan treatment for 1 h. Click here for file

Additional file 7: Figure S3 Construction of BLRP-MKL1-BirA stable cell lines. Click here for file

Additional file 8: Table S5 SRF binding peaks in BLRP-MKL1-BirA stable cells (tag count more than 20). Click here for file

Additional file 9: Table S6 MKL1 binding peaks in BLRP-MKL1-BirA stable cells (tag count more than 20). Click here for file

Additional file 10: Table S7 Elk4 binding peaks in BLRP-MKL1-BirA stable cells (tag count more than 20). Click here for file

Additional file 11: Table S8 The 316 ncRNAs associated with SRF intergenic peaks. Click here for file

Additional file 12: Table S9 Part I: Gene Ontology results (Biological process annotations, p < 0.05) for SRF peak associated genes (tag count > 20, in zymosan treated BLRP-MKL1-BirA cells). Part II: Gene Ontology results (Biological process annotations, p < 0.05) for MKL1 peak associated genes (tag count > 20, in zymosan treated BLRP-MKL1-BirA cells). Part III: Gene Ontology results (Biological process annotations, p < 0.05) for ELK4 peak associated genes (tag count > 20, in zymosan treated BLRP-MKL1-BirA cells). Click here for file

Additional file 13: Table S10 Part I: KEGG pathway analysis full results (p < 0.05) for SRF peak associated genes (tag count > 20, in zymosan treated BLRP-MKL1-BirA cells). Part II: KEGG pathway analysis full results (p < 0.05) for MKL1 peak associated genes (tag count > 20, in zymosan treated BLRP-MKL1-BirA cells). Part III: KEGG pathway analysis full results (p < 0.05) for ELK4 peak associated genes (tag count > 20, in zymosan treated BLRP-MKL1-BirA cells). Click here for file
