# Supplementary material for: MKL1/2 and ELK4 co-regulate distinct serum response factor (SRF) transcription programs in macrophages
Source: BMC Genomics. 2014 Apr 23;15:301. doi: 10.1186/1471-2164-15-301 (PMC4023608; doi:10.1186/1471-2164-15-301)
Supplement: Additional file 1: Figure S1 — SRF functions in the early phase of zymosan induction in macrophages. [file 1471-2164-15-301-S1.pdf]

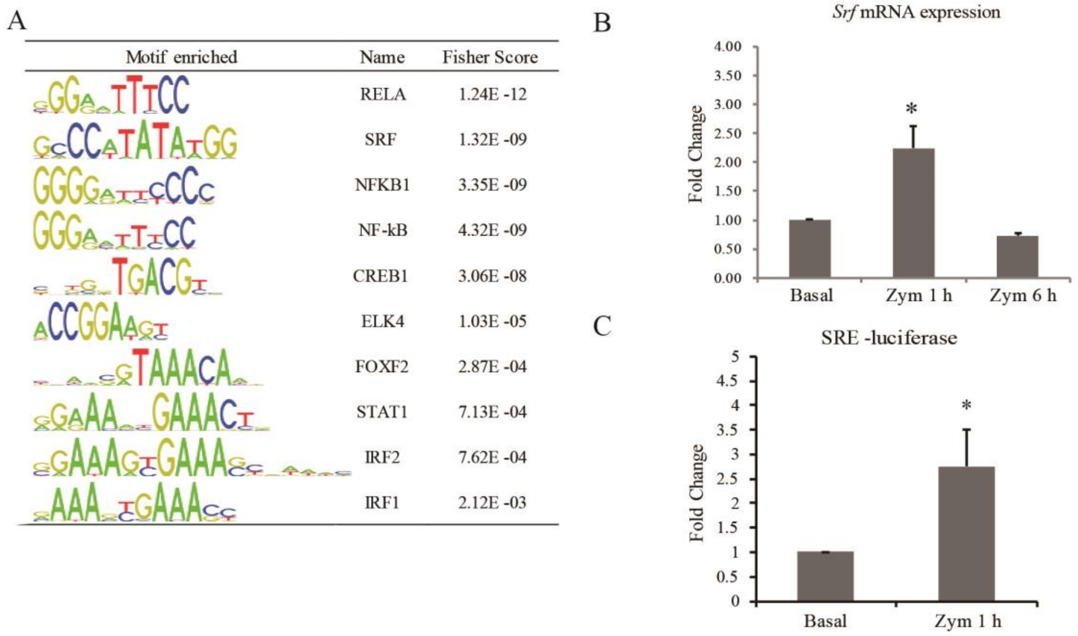

Supplemental Figure 1 SRF functions in the early phase of zymosan induction in macrophages

(A) Logos of sequence motifs over-represented in the promoters of genes induced > 2-fold in elicited macrophages following zymosan treatment for 1h. Relative height of letters indicates the frequency of occurrence at each position. Binding sites for SRF and ELK4 are among the most highly enriched sequence motifs in the promoters of this set of genes. (B) mRNA expression for *Srf* at 0, 1 and 6 h after zymosan (1 mg/ml) treatment in thioglycollate-elicited, peritoneal macrophages. (C) RAW264.7 cells were transiently transfected with a 3XCArG directing luciferase reporter plasmid, untreated or treated with zymosan for 1 h. Relative luciferase activity was shown as fold change to untreated condition.
