# Supplementary material for: MKL1/2 and ELK4 co-regulate distinct serum response factor (SRF) transcription programs in macrophages
Source: BMC Genomics. 2014 Apr 23;15:301. doi: 10.1186/1471-2164-15-301 (PMC4023608; doi:10.1186/1471-2164-15-301)
Supplement: Additional file 4: Figure S2 — Knockdown of MKL1/2 and ELK4 has an impact on zymosan treated macrophages. [file 1471-2164-15-301-S4.pdf]

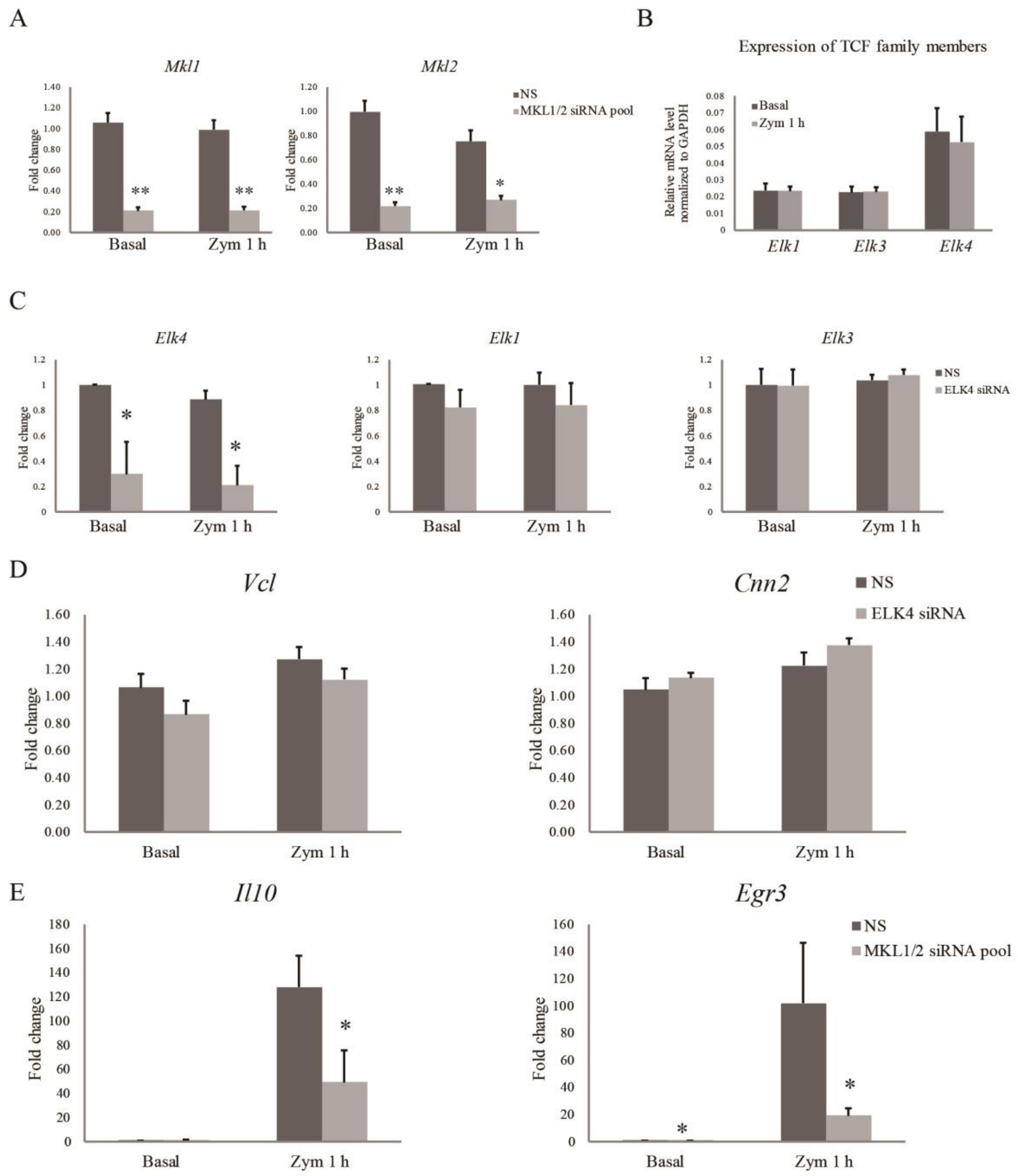

Supplemental Figure 2 Knockdown of MKL1/2 and ELK4 has an impact on zymosan treated macrophages

(A) The efficiency of MKL1/2 double knockdown was confirmed by Q-PCR. Thioglycollate-elicited macrophages were transfected with control or pooled siRNAs targeting MKL1 and MKL2, without or with 1 h of zymosan treatment as indicated, and the fold change of *Mkl1* and *Mkl2* mRNA compared to non-specific(NS) siRNA

transfected samples are shown. (B) Expression level of TCF family members *Elk1*, *Elk3* and *Elk4* in primary macrophages under basal condition and after zymosan treatment of 1 h. (C) ELK4 knockdown efficiency and specificity was confirmed by Q-PCR. Thioglycollate-elicited macrophages were transfected with control or ELK4 specific siRNAs, without or with 1 h of zymosan treatment, and the fold change of *Elk1*, *Elk3* and *Elk4* mRNA compared to NS siRNA transfected samples are shown. (D) mRNA expression levels of classical MKL1/2 target genes *Vcl* and *Cnn2* were analyzed in ELK4 knockdown samples. Thioglycollate-elicited macrophages were transfected with nonspecific (NS) or specific siRNAs for ELK4 under basal conditions and after 1 h of zymosan treatment. (E) mRNA expression levels of classical ELK4 target genes *Il10* and *Egr3* were analyzed in MKL1/2 double knockdown samples. Thioglycollate-elicited macrophages were transfected with nonspecific (NS) or siRNAs pool for MKL1 and MKL2 under basal conditions and after 1 h of zymosan treatment. **$p < 0.01$ vs NS; *$p < 0.05$ vs NS.
