# Supplementary material for: MKL1/2 and ELK4 co-regulate distinct serum response factor (SRF) transcription programs in macrophages
Source: BMC Genomics. 2014 Apr 23;15:301. doi: 10.1186/1471-2164-15-301 (PMC4023608; doi:10.1186/1471-2164-15-301)
Supplement: Additional file 7: Figure S3 — Construction of BLRP-MKL1-BirA stable cell lines. [file 1471-2164-15-301-S7.pdf]

A

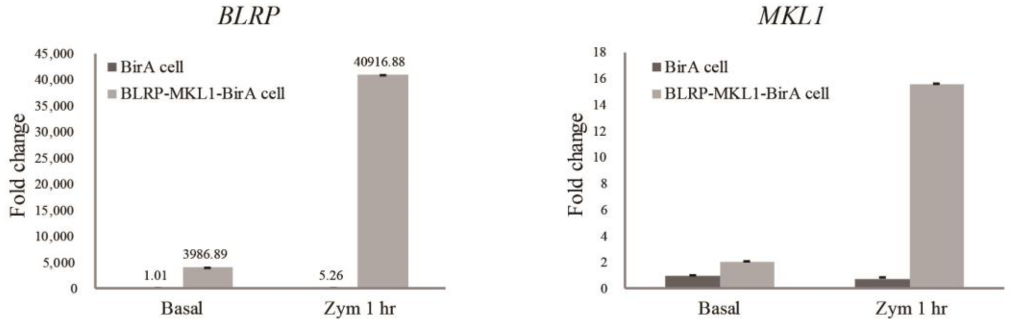

B

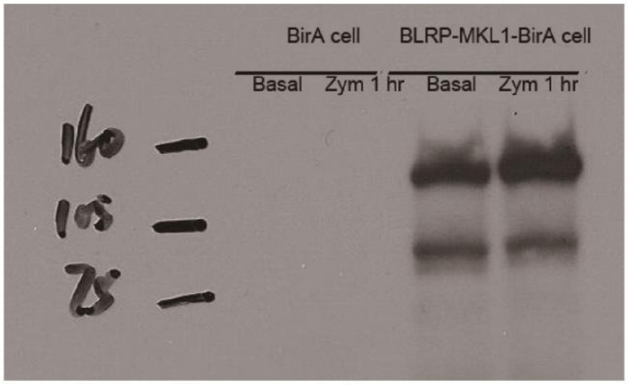

Supplemental Figure 3 Construction of BLRP-MKL1-BirA stable cell lines

(A) and (B) mRNA expression levels of BLRP and MKL1 was analyzed by Q-PCR, respectively. The expression levels of BLRP and MKL1 in BLRP-MKL1-BirA stable cell lines were compared to those in BirA parent cell lines, without or with zymosan treatment for 1 h. (C) Western blots for MKL1 were performed in BLRP-MKL1-BirA stable cell lines and the BirA parent cell lines.
